# Natural variation in a molybdate transporter confers salt tolerance in tomato

**DOI:** 10.1093/plphys/kiaf004

**Published:** 2025-01-07

**Authors:** Zhen Wang, Yechun Hong, Zhaojun Guo, Dianjue Li, Zhen-Fei Chao, Guangtao Zhu, Jian-Kang Zhu

**Affiliations:** School of Life Sciences, Anhui Agricultural University, Hefei 230036, China; Institute of Advanced Biotechnology and School of Medicine, Southern University of Science and Technology, Shenzhen 518055, China; School of Life Sciences, Anhui Agricultural University, Hefei 230036, China; The AGISCAAS-YNNU Joint Academy of Potato Sciences, Yunnan Normal University, Kunming 650500, China; Center for Excellence in Molecular Plant Sciences, Chinese Academy of Sciences, Shanghai 200032, China; The AGISCAAS-YNNU Joint Academy of Potato Sciences, Yunnan Normal University, Kunming 650500, China; Institute of Advanced Biotechnology and School of Medicine, Southern University of Science and Technology, Shenzhen 518055, China

## Abstract

Natural variation in a molybdate transporter-encoding gene is associated with molybdenum accumulation, which reduces hypocotyl growth and improves salt tolerance in tomato.

Dear Editor,

Excessive fertilization and freshwater scarcity extremely accelerate salt salinization around the world (Hassani et al. 2021). Salt stress caused by soil salinization impairs plant growth and development by disrupting cellular ion homeostasis, thus seriously threatening crop productivity and food security (Colin et al. 2023). In the process of long-term adaptation to salt stress, plants have evolved natural alleles residing in genetic loci to maintain ion homeostasis (Zhu 2016; Yang and Guo 2018). Therefore, investigating natural alleles associated with ion variations contributes to elucidate how plants adapt to salt stress. Molybdenum (Mo) is an essential trace element for plants growth and development, which enters into the active sites of key enzymes involved in the metabolism of carbon, nitrogen, and sulfur in the form of Mo cofactors (Schwarz et al. 2009; Huang et al. 2019, 2022). However, the natural alleles controlling Mo accumulation are still largely unknown in tomato.

To understand genetic basis of Mo accumulation in tomato response to salt stress, we determined the Mo concentration in the shoots and roots of 365 tomato accessions, including 34 wild *Solanum pimpinellifolium*, 118 domesticated *Solanum lycopersicum* var. *cerasiforme*, and 213 improved cultivars *S. lycopersicum*, under salt stress conditions (Supplementary Data Set S1). We subsequently conducted genome-wide associated studies (GWAS) for Mo content and discovered a major signal strongly associated with shoot Mo accumulation under salt stress (Fig. 1A; Supplementary Fig. S1). A homolog of *Arabidopsis MOLYBDATE TRANSPORTER 1* (*MOT1*) was identified in this signal and named *SlMOT1* (Solyc10g084680), which harbors a 3-bp indel (namely indel595) located 595-bp downstream of the translation start codon (Supplementary Data Set S2). Based on this variation, 354 tomato accessions were divided into haplotype 1 (Hap1) group representing the reference sequence and Hap2 with 3-bp insertion at indel595 that resulted in an extra aspartic acid (D) at position 199 in the extracellular segment of SlMOT1 (Fig. 1B; Supplementary Data Set S3). The accumulation of Mo in shoots of Hap1 varieties was clearly lower than that of Hap2 accessions, while the concentration of Mo in roots had no significant difference between the 2 haplotypes (Fig. 1, C and D), further supporting that the indel595 is closely associated with Mo accumulation in shoots rather than in roots under salt stress.

**Figure 1.**
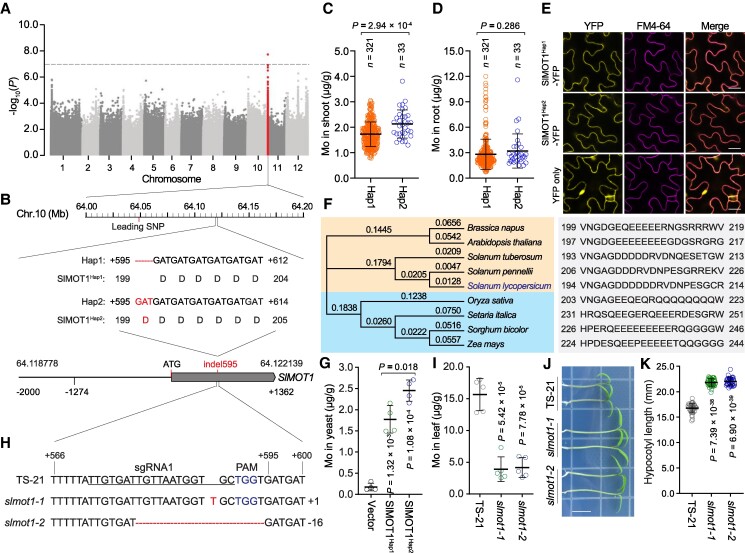
Identification and genetic validation of *MOT1* in tomato. **A)** Manhattan plot showing GWAS result of shoot Mo concentration under salt stress. The associated SNPs are highlighted in red. Dashed line indicates the significance threshold. **B)** Gene model of *SlMOT1* and the physical distance between indel595 and the leading SNP. The nucleotide and amino acid sequences of the 2 alleles are shown. Black box and line represent coding sequence and UTR, respectively. **C, D)** Comparison of Mo contents in shoots **C)** and roots **D)** of the 2 haplotype groups, Hap1 and Hap2. *n* indicates the number of accessions belonging to each haplotype. **E)** Subcellular localization of the 2 SlMOT1 variants in tomato leaves. FM4-64 is a plasma membrane marker. Scale bar, 20 *μ*m. **F)** Phylogenic tree of MOT1 homologs was generated using a MEGA program. The amino acid sequences show variations in the second extracellular region of MOT1 proteins. **G)** Molybdate transport activities of 2 SlMOT1 variants in yeast (*n* = 4 biological replicates). **H)** Nucleotide sequences of *SlMOT1* in wild-type TS-21, *slmot1-1*, and *slmot1-2*. Deletion and insertion are highlighted in red. **I)** Determination of Mo content in leaves of wild-type TS-21 and *slmot1* mutants (*n* = 5 biological replicates). **J, K)** The elongation of hypocotyls in 5-d-old wild-type TS-21 and *slmot1* mutants under normal conditions (*n* = 38 seedlings for each genotype). Scale bar, 1 cm. In **C)**, **D)**, **G)**, **I)**, and **K)**, *P*-values were determined by Student's *t*-test; error bars represent means ± Sd. PAM, protospacer adjacent motif; SNP, single nucleotide polymorphism; YFP, yellow florescence protein.

Subcellular localization analysis indicated that the additional aspartic acid has no effect on the plasma membrane localization of SlMOT1 in tomato (Fig. 1E). Phylogenetic tree revealed that MOT1 is evolutionarily conserved in both monocots and dicots, especially its second extracellular segment rich in polar acidic amino acids (Fig. 1F). Since Mo is generally taken up and transported as molybdate through MOT1 in plants (Huang et al. 2022), we speculated that these polar acidic amino acids might contribute to the binding affinity of molybdate. Our data showed that the second extracellular segment of SlMOT^Hap2^ is higher affinity for binding molybdate than that of SlMOT1^Hap1^ in vitro, while the segment of AtMOT1 containing more polar acidic amino acids exhibited the highest affinity for molybdate among the tested segments (Supplementary Fig. S2). Moreover, we found that the molybdate transport activity of SlMOT^Hap2^ was evidently higher than that of SlMOT1^Hap1^ in yeast (Fig. 1G). These results suggested that the extra aspartic acid caused by this variation in SlMOT1 likely enhances molybdate transport activity, which contributes to Mo accumulation in tomato.

To characterize the function of *SlMOT1*, 2 mutant alleles (namely *slmot1-1* and *slmot1-2*) were generated by using CRISPR/Cas9 system in a Hap2 accession TS-21 (Fig. 1H). These 2 *slmot1* mutants exhibited reduced Mo concentration and enhanced hypocotyl elongation (Fig. 1, I to K), suggesting that SlMOT1-mediated Mo accumulation is crucial for tomato development. Given that cellular Mo accumulation contributes to phytohormone biosynthesis and metabolism (Huang et al. 2022; Zhang et al. 2023). We speculated that hypocotyl elongation may be affected by auxin or cytokinin accumulation in *slmot1* mutants. Interestingly, phenotype analysis indicated that *slmot1* mutants are clearly decreased in salt tolerance (Fig. 2, A to C; Supplementary Fig. S3). Two *SlMOT1^Hap1^* mutant alleles generated by CRISPR/Cas9 in Ailsa Craig (AC) also exhibited hypersensitive phenotype under salt stress (Fig. 2, E to H), supporting that the 2 natural alleles of *SlMOT1* are involved in tomato response to salt stress. The Mo cofactor is required for the activity of aldehyde oxidase 3 that catalyzes the final step in abscisic acid (ABA) biosynthesis in *Arabidopsis* (Xiong et al. 2001; Mendel and Hänsch 2002). Our data showed that the upregulated level of ABA in *slmot1* mutants is significantly lower than that in wild type after salt treatment (Fig. 2D). A dramatic increase in ABA biosynthesis is caused by salt stress in the shoot, which reduces stomatal conductance through promoting stomatal closure (Hedrich and Shabala 2018). We found that the stomatal conductance of *slmot1* mutants is clearly higher than that of wild type (Fig. 2I), which is consistent with lower ABA accumulation in *slmot1* mutants under salt stress. Root-to-shoot translocation of sodium (Na^+^) is affected by stomatal conductance, thus resulting in disruption of Na^+^ and potassium (K^+^) homeostasis in plants under salt stress (Karimi et al. 2021). Ion content analysis revealed that *slmot1* mutants accumulated more Na^+^ but similar levels of K^+^ in leaves when compared with wild-type plants after treatment with 100 mm sodium chloride (NaCl), whereas *slmot1* mutants showed lower levels of K^+^ but accumulated notably higher Na^+^ in leaves compared with wild type after treatment with 150 mm NaCl, thus causing a significantly higher Na^+^/K^+^ ratio in *slmot1* mutant leaves than in wild-type leaves (Fig. 1, J to L). These results suggest that Mo accumulation caused by SlMOT1 is critical for ABA biosynthesis, which affects stomatal conductance and leaf Na^+^/K^+^ homeostasis, contributing to salt tolerance in tomato. Overall, association analysis of Mo accumulation provides insight into the genetic basis of salt tolerance in tomato.

**Figure 2.**
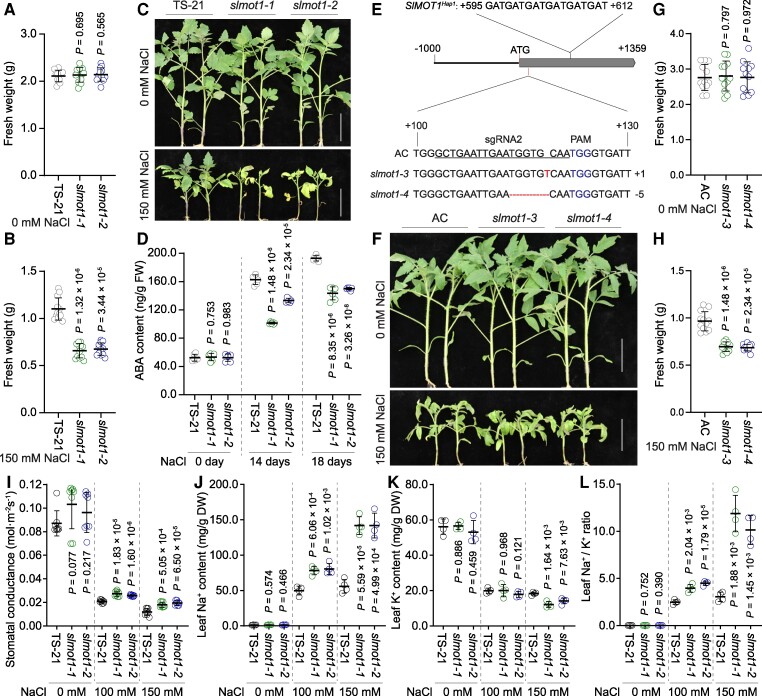
*SlMOT1* contributes to ABA accumulation and salt tolerance in tomato. **A** to **C)** Salt tolerance assay of *SlMOT1^Hap2^* mutants. Two-week-old wild-type TS-21, *slmot1-1*, and *slmot1-2* grown in medium containing 0 or 150 mm NaCl for 10 d (*n* = 12 plants for each genotype). Scale bars, 5 cm. **D)** ABA content was measured in wild-type TS-21, *slmot1-1*, and *slmot1-2* plants after treatment with 100 mm NaCl for indicated days (*n* = 6 replicates for each genotype). **E)** Nucleotide sequences of *SlMOT1* in wild-type AC, *slmot1-3*, and *slmot1-4*. Black box and line represent coding sequence and promoter region, respectively. The insertion and deletion are marked in red. **F** to **H)** Salt tolerance assay of *SlMOT1^Hap1^* mutants. Two-week-old AC, *slmot1-3*, and *slmot1-4* grown in medium containing 0 or 150 mm NaCl for 7 d (*n* = 12 plants for each genotype). Scale bars, 5 cm. **I)** Measurement of stomatal conductance of 14-d-old wild-type, *slmot1-1*, and *slmot1-2* plants after treatment with 0, 100, or 150 mm NaCl for 10 d (*n* = 8 plants for each genotype). **J** to **L)** Na^+^ and K^+^ contents and Na^+^/K^+^ ratio in leaves of 14-d-old wild-type TS-21 and *slmot1* mutants after treatment with 0, 100, or 150 mm NaCl for 10 d (*n* = 4 biological replicates). In **A)**, **B)**, **D)**, and **G)** to **L)**, data are means ± Sd; *P*-values were determined by Student's *t*-test. PAM, protospacer adjacent motif.

## Supplementary Material

kiaf004_Supplementary_Data

## Data Availability

All data generated or analyzed during this study are included in this published article and its supplementary information files.

## Supplementary data

The following materials are available in the online version of this article.

**Supplementary Figure S1.** GWAS for Mo concentration in tomato roots.

**Supplementary Figure S2.** The binding affinity of Mo transporters.

**Supplementary Figure S3.** *SlMOT1* is involved in tomato salt tolerance.

**Supplementary Table S1.** Primer sequences used in this study.

**Supplementary Data Set S1.** The Mo concentration in the shoots and roots of 365 tomato accessions under salt stress.

**Supplementary Data Set S2.** Gene information in shoot Mo concentration-associated sweeps.

**Supplementary Data Set S3.** Distribution of indel595 in the tomato population.

## Dive Curated Terms

The following phenotypic, genotypic, and functional terms are of significance to the work described in this paper:

mot1 Gramene: AT2G25680
mot1 Araport: AT2G25680

ABA Gramene: Abscisic acid
ABA Araport: Abscisic acid

MEGA CHEBI: CHEBI:6617

MOLYBDATE CHEBI: CHEBI:36264

ion CHEBI: CHEBI:24870

molybdenum CHEBI: CHEBI:28685

salt CHEBI: CHEBI:24866

## References

[kiaf004-B1] Colin L , Ruhnow F, Zhu JK, Zhao CZ, Zhao Y, Persson S. The cell biology of primary cell walls during salt stress. Plant Cell. 2023:35(1):201–217. 10.1093/plcell/koac292 36149287 PMC9806596

[kiaf004-B2] Hassani A , Azapagic A, Shokri N. Global predictions of primary soil salinization under changing climate in the 21st century. Nat Commun. 2021:12(1):6663. 10.1038/s41467-021-26907-3 34795219 PMC8602669

[kiaf004-B3] Hedrich R , Shabala S. Stomata in a saline world. Curr Opin Plant Biol. 2018:46:87–95. 10.1016/j.pbi.2018.07.015 30138845

[kiaf004-B4] Huang XY , Hu DW, Zhao FJ. Molybdenum: more than an essential element. J Exp Bot. 2022:73(6):1766–1774. 10.1093/jxb/erab534 34864981

[kiaf004-B5] Huang XY , Liu H, Zhu YF, Pinson SRM, Lin HX, Guerinot ML, Zhao FJ, Salt DE. Natural variation in a molybdate transporter controls grain molybdenum concentration in rice. New Phytol. 2019:221(4):1983–1997. 10.1111/nph.15546 30339276

[kiaf004-B6] Karimi SM , Freund M, Wager BM, Knoblauch M, Fromm J, Mueller HM, Ache P, Krischke M, Mueller MJ, Müller T, et al Under salt stress guard cells rewire ion transport and abscisic acid signaling. New Phytol. 2021:231(3):1040–1055. 10.1111/nph.17376 33774818

[kiaf004-B7] Mendel RR , Hänsch R. Molybdoenzymes and molybdenum cofactor in plants. J Exp Bot. 2002:53(375):1689–1698. 10.1093/jxb/erf038 12147719

[kiaf004-B8] Schwarz G , Mendel RR, Ribbe MW. Molybdenum cofactors, enzymes and pathways. Nature. 2009:460(7257):839–847. 10.1038/nature08302 19675644

[kiaf004-B9] Xiong LM , Ishitani M, Lee H, Zhu JK. The Arabidopsis locus encodes a molybdenum cofactor sulfurase and modulates cold stress- and osmotic stress-responsive gene expression. Plant Cell. 2001:13(9):2063–2083. 10.1105/tpc.010101 11549764 PMC139452

[kiaf004-B10] Yang YQ , Guo Y. Unraveling salt stress signaling in plants. J Integr Plant Biol. 2018:60(9):796–804. 10.1111/jipb.12689 29905393

[kiaf004-B11] Zhang J , Liu SL, Liu CB, Zhang M, Fu XQ, Wang YL, Song T, Chao ZF, Han ML, Tian ZX, et al Natural variants of molybdate transporters contribute to yield traits of soybean by affecting auxin synthesis. Curr Biol. 2023:33(24):5355–5367.e5. 10.1016/j.cub.2023.10.072 37995699

[kiaf004-B12] Zhu JK . Abiotic stress signaling and responses in plants. Cell. 2016:167(2):313–324. 10.1016/j.cell.2016.08.029 27716505 PMC5104190

