# Supplementary material for: Natural variation in a molybdate transporter confers salt tolerance in tomato
Source: Plant Physiol. 2025 Jan 7;197(2):kiaf004. doi: 10.1093/plphys/kiaf004 (PMC11831802; doi:10.1093/plphys/kiaf004)
Supplement: kiaf004_Supplementary_Data [file kiaf004_supplementary_data.zip › PP2024LTR02546R1_Supplemental_Figures.pdf]

Supplementary Figures

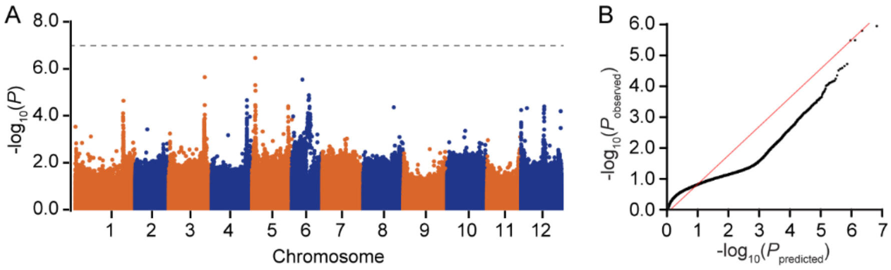

Supplementary Figure S1 GWAS for molybdenum concentration in tomato roots.

(A) Manhattan plot showing the GWAS result of the concentrations of molybdenum in roots of 365 tomato accessions after salt stress treatment. The dashed line indicates the Bonferroni-adjusted significance threshold. (**B**) The Q-Q plots of the GWAS for root molybdenum contents in 365 tomato accessions after salt stress treatment. The red line and black dots represent the distribution of predicted and observed values, respectively.

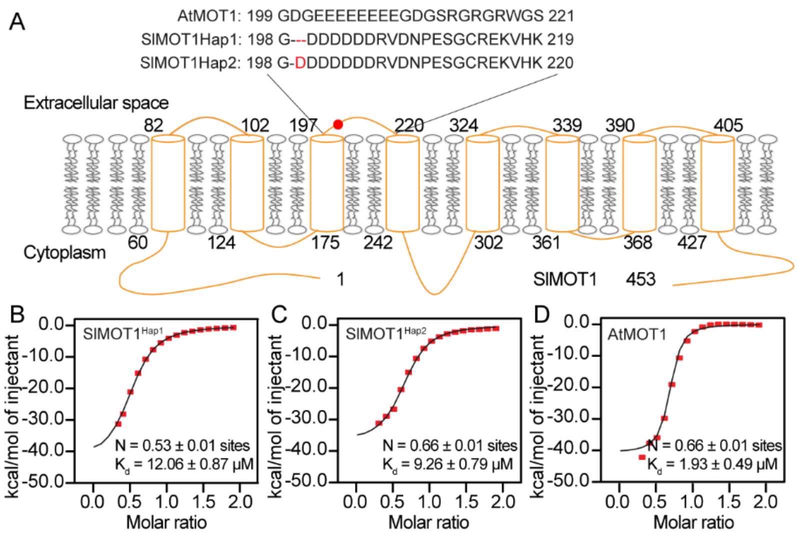

Supplementary Figure S2 The binding affinity of molybdenum transporters.
(A) Schematic representation of the predicted structure of SlMOT1 and amino acid changes caused by indel595 in the two haplotypes. The amino acid changes were highlighted in red. The amino acid sequence of the second extracellular fragment in AtMOT1 was shown as a control. The predicted transmembrane helices are shown as cylinders through the lipid bilayer. **(B - D)** Determination of the affinity of the second extracellular region of MOT1 proteins bound to molybdate by the isothermal titration calorimetry (ITC). The theoretical curve was fitted to the integrated data using a single-site model in these cases.

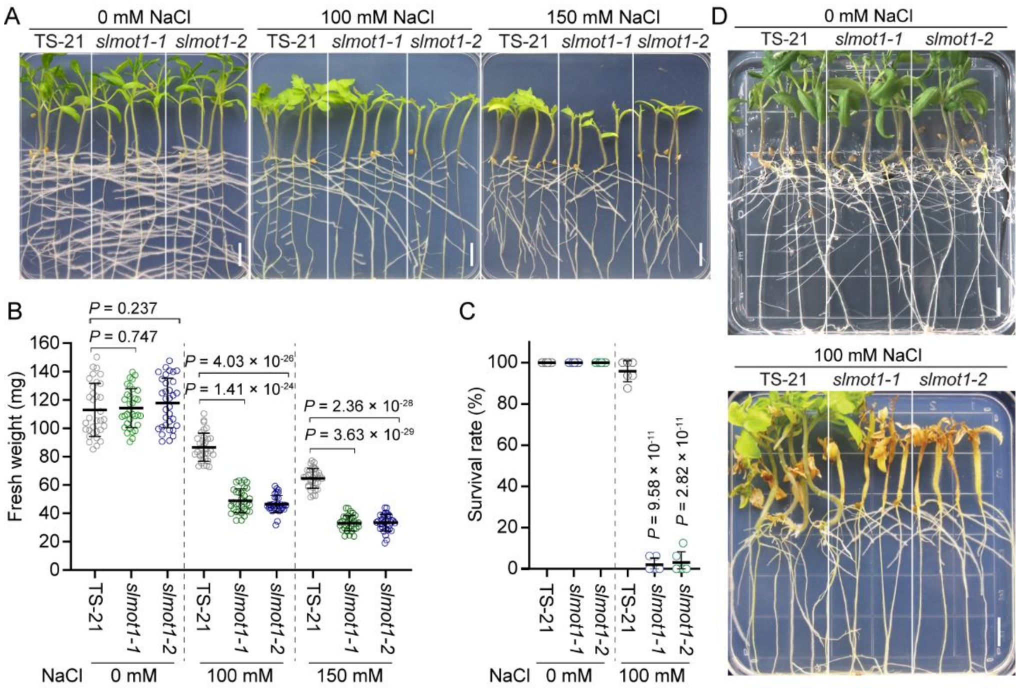

Supplementary Figure S3 *SlMOT1* is involved in tomato salt tolerance.
(A) Photographs of 5-day-old wild type TS-21 and *slmot1* mutant plants grown in medium containing 0, 100, or 150 mM NaCl for 14 days. Scale bars, 1 cm. **(B)** Fresh weights of plants shown in (A). Values represent the means ± SD (n = 33 plants of each genotype). **(C)** Survival rate of plants shown in (D). Error bars are the means ± SD (n = 6 biological replicates). **(D)** Salt tolerance assay of wild type and *slmot1* mutants. Seven-day-old wild-type (TS-21), *slmot1-1*, and *slmot1-2* plants were subjected to high salinity (100 mM NaCl) for 4 weeks. Plants grown under normal conditions for 18 days were used as control. Scale bars, 1 cm. In (B and C), the *P*-values were determined by using a Student's *t*-test.

Supplementary Table S1. List of primer sequences used in this study

**Name**	**Primer sequence**	**Description**
sgRNA1-FP	TGATT GTTGTGATTGTTAATGGTGC	sgRNAs for *SlMOT1*
sgRNA1-RP	AAACGCACCATTAACAATCACAACA	
sgRNA2-FP	TGATTGGCTGAATTGAATGGTGCAA	
sgRNA2-RP	AAACTTGCACCATTCAATTCAGCCA	
SlMOT1-seqFP	TGCAAGTCAACTGCTACCTCT	Sequencing for *slmot1* mutations
SlMOT1-seqrP	AACCCTTGCTTCCAAGCATGT	
SlMOT1-indel-FP	TGCAAGTCAACTGCTACCTCT	Genotyping for indel595
SlMOT1-indel-RP	AACCCTTGCTTCCAAGCATGT	
SlMOT1-YFP-FP	GAATTCATGGAGTCCTCCACACTAGAA	*35S:SlMOT1-YFP*
SlMOT1-YFP-RP	GTCGACTGGGTTTCTATGAAACCAAATTAC	

Supplementary Materials and Methods

Plant material and growth conditions

All tomato seeds used in this study were obtained from the IVF-CAAS (Institute of Vegetables and Flowers, Chinese Academy of Agricultural Science) and list in the Supplementary Dataset S1. Tomato seeds were surface-sterilized and sown in 0.25× MS medium (pH 6.0) with 0.6% (w/v) agar to germinate at 23°C. Ten-day-old seedlings were then transplanted to soil and cultivate in a growth room at 25°C with 16-h-light / 8-h-dark period for seeds proliferation.

Determination of ion contents

The samples were collected for ion analysis as described previously (Chao et al., 2013). Briefly, 19-day-old tomato seedlings were grown in 0.25× Hoagland liquid medium with 150 mM NaCl for 1 d in a growth room. The shoots and roots were rinsed three times with 10 mM EDTA to eliminate any contaminants and dehydrated in an oven at 65°C before digesting in 1 mL nitric acid containing an indium (In) internal standard at 115°C for 3 h. The digested samples were diluted to 10 mL with deionized water and determined ion content by using an inductively coupled plasma mass spectrometry (ICP-MS) (NexION 350D; PerkinElmer) coupled to an Apex desolation system and an SC-4 DX auto sampler (Elemental Scientific Inc., Omaha, NE, US). The molybdenum, sodium, and potassium contents were finally normalized in calculation as determined using a heuristic algorithm based on the weights of 12 weighed dry samples and the solution concentrations.

Genome-wide association studies

The genomic resequencing of all 365 tomato accessions were devoted to construct the association panel as described previously (Zhu et al., 2018). GWAS for the trait was executed by using 2,824,130 SNPs across the whole tomato genome via utilizing a FaST-LMM program with minor allele frequency ≥ 0.05 and missing ratio < 10%. A strict P value threshold of 8.45×10^{-8} was set as the significance threshold at the whole genome level. The most significant variations within 0.5 Mb interval of

association were identified as the candidates of leading natural variations.

Generation of knockout mutants.

The CRISPR-Cas9 system was conducted to generate knockout mutants as described previously (Hong et al., 2023). Single guide RNA (sgRNA) nearby a protospacer-adjacent motif (PAM) sequence was cloned into a binary vector harboring the Cas9 expression cassette, which is controlled by an *Arabidopsis thaliana U6* promoter. The constructs were then introduced into a wild tomato accession TS-21 and a modern tomato cultivar Ailsa Craig (AC) to generate mutant alleles. The homozygous mutants were identified from T_1 generation by PCR-based sequencing. The primers are listed in Supplementary Table S1.

Determination of the binding affinity

The binding affinity of MOT1 and molybdate was determined by isothermal titration calorimetry (ITC) as described previously (Quinn et al., 2016). The second extracellular fragments of $SlMOT1^{Hap1}$, $SlMOT1^{Hap2}$, and AtMOT1 shown in Supplementary Figure S2A were synthesized and diluted in a binding buffer containing 20 mM HEPES (pH7.0), 100 mM NaCl, and 2 mM *β*-mercaptoethanol to a concentration of about 0.8 - 0.12 mM. The sodium molybdate dihydrate was also dissolved in this buffer with a concentration of about 1.0 - 1.5 mM. The binding affinity was measured by ITC using an automated MicroCal PEAQ-ITC (Malvern Panalytical) and the data were then analyzed through the MicroCal Origin 7.0 program. The area of each peak was plotted versus the molar ratio of molybdate to peptide that gives us the reaction stoichiometry, and then the affinity (K_d) was derived from the fitted binding curve.

Molybdate transport assay

The p416GPD-empty vector, p416GPD-$SlMOT1^{Hap1}$, and p416GPD-$SlMOT1^{Hap2}$ were introduced into AH109 strains of *S. cerevisiae*. Positive transformants were confirmed by using PCR analysis after selection on Ura-deficient medium. Yeast cells

were cultured in molybdate-free liquid medium for 1 day, and then exposed to a 200 nM molybdate-supplemented liquid medium for 2 hours. Subsequently, the yeast cells were rinsed with ice-cold deionized water for 5 times, and then collected for ion analysis by ICP-MS.

Physiological analysis

For salt tolerance assay, tomato seedlings grown in 0.25× Hoagland medium or soil under a long-day period (16-h-light / 8-h-dark) were treated with different concentration of sodium chloride. The biomass and survival rate were measured at the end of experiments.

Measurement of stomatal conductance

The third fully emerged leaves were selected to measure stomatal conductance using the Li-6400*XT* portable photosynthesis system (Li-COR Biosciences) as described previously (Kaiser et al., 2020; Rasouli et al., 2021). Conditions inside the leaf chamber were set to 500 μmol $m^{-2}s^{-1}$, the CO_2 concentration at 520 ppm, air temperature at 23°C, and relative humidity at 30-35%. Eight plants per genotype were measured in this experiment.

Statistical analysis

Statistical data were processed in Microsoft Excel 2019, and the images were generated by using Graphpad Prism (v.8.0, GraphPad, San Diego, CA, USA). All sets of data groups in this study were determined by using Student's t-test. Differences were considered statistically significant at $P < 0.05$.

References

Chao DY, Dilkes B, Luo HB, Douglas A, Yakubova E, Lahner B, Salt DE (2013) Polyploids Exhibit Higher Potassium Uptake and Salinity Tolerance in. Science **341:** 658-659

Hong YC, Guan XJ, Wang X, Kong DL, Yu SJ, Wang ZQ, Yu YD, Chao ZF, Liu

X, Huang SW, Zhu JK, Zhu GT, Wang Z (2023) Natural variation in SlSOS2 promoter hinders salt resistance during tomato domestication. Horticulture Research **10:** uhac244

Kaiser E, Morales A, Harbinson J, Heuvelink E, Marcelis LFM (2020) High Stomatal Conductance in the Tomato Mutant Allows for Faster Photosynthetic Induction. Frontiers in Plant Science **11:** 1317

Quinn CF, Carpenter MC, Croteau ML, Wilcox DE (2016) Isothermal Titration Calorimetry Measurements of Metal Ions Binding to Proteins. Calorimetry **567:** 3-21

Rasouli F, Kiani-Pouya A, Tahir A, Shabala L, Chen ZH, Shabala S (2021) A comparative analysis of stomatal traits and photosynthetic responses in closely related halophytic and glycophytic species under saline conditions. Environmental and Experimental Botany **181:** 104300

Zhu GT, Wang SC, Huang ZJ, Zhang SB, Liao QG, Zhang CZ, Lin T, Qin M, Peng M, Yang CK, Cao X, Han X, Wang XX, van der Knaap E, Zhang ZH, Cui X, Klee H, Fernie AR, Luo J, Huang SW (2018) Rewiring of the Fruit Metabolome in Tomato Breeding. Cell **172:** 249-261
